# Novel sofosbuvir derivatives against SARS-CoV-2 RNA-dependent RNA polymerase: an in silico perspective

**DOI:** 10.1038/s41598-023-49712-y

**Published:** 2023-12-27

**Authors:** Abdulwahed Alrehaily, Abdo A. Elfiky, Ibrahim M. Ibrahim, Mohamed N. Ibrahim, Amr Sonousi

**Affiliations:** 1 Biology Department, Faculty of Science, Islamic University of Madinah, 42351 Madinah, Saudi Arabia; 2 Biophysics Department, Faculty of Science, Cairo University, Giza, Egypt; 3 Clinical Laboratories Department, College of Applied Medical Sciences, Jouf University, Qurrayat, Saudi Arabia; 4 Pharmaceutical Organic Department, Faculty of Pharmacy, Cairo University, Giza, Egypt; 5 University of Hertfordshire Hosted By Global Academic Foundation, New Administrative Capital, Cairo, Egypt

**Keywords:** Biophysics, Computational biology and bioinformatics

## Abstract

The human coronavirus, SARS-CoV-2, had a negative impact on both the economy and human health, and the emerging resistant variants are an ongoing threat. One essential protein to target to prevent virus replication is the viral RNA-dependent RNA polymerase (RdRp). Sofosbuvir, a uridine nucleotide analog that potently inhibits viral polymerase, has been found to help treat SARS-CoV-2 patients. This work combines molecular docking and dynamics simulation (MDS) to test 14 sofosbuvir-based modifications against SARS-CoV-2 RdRp. The results reveal comparable (slightly better) average binding affinity of five modifications (compounds **3**, **4**, **11**, **12**, and **14**) to the parent molecule, sofosbuvir. Compounds **3** and **4** show the best average binding affinities against SARS-CoV-2 RdRp (− 16.28 ± 5.69 and − 16.25 ± 5.78 kcal/mol average binding energy compared to − 16.20 ± 6.35 kcal/mol for sofosbuvir) calculated by Molecular Mechanics Generalized Born Surface Area (MM-GBSA) after MDS. The present study proposes compounds **3** and **4** as potential SARS-CoV-2 RdRp blockers, although this has yet to be proven experimentally.

## Introduction

SARS-CoV-2 has infected an estimated 769 million people, with about 6.9 million deaths since its outbreak in 2019 (https://www.who.int/emergencies/diseases/novel-coronavirus-2019). The disease symptoms can range from mild symptoms such as cough, fever, fatigue, loss of smell, and loss of taste to more severe complications such as severe pneumonia, dyspnea, and organ dysfunction^1^. SARS-CoV-2 (also known as Covid-19) is a single-stranded RNA virus in the Coronaviridae family^2^. SARS-CoV-2 has many known variants, such as alpha, beta, gamma, delta, and omicron variant^3^. In addition, other variants of interest include, epsilon, zeta, eta, theta, iota, kappa, lambda, and mu. Although disease mortality and morbidity have decreased with the development of vaccination, the emergence of different variants able to overcome the vaccines has caused recent waves and breakthrough infections^4^.

Several viral targets have been used in COVID-19 drug development, and these encompass crucial stages of the virus replication cycle^5–9^. For example, the spike protein is necessary during viral recognition and host-cell entry, the main protease (M^pro^) during the proteolytic activation stage, and RNA-dependent RNA polymerase (RdRp) during the transcription stage^5^. Of these targets, RdRp has been shown to be the most appealing for drug discovery as it is a very conservative non-structural protein functions as RNA polymerase, which either copies the RNA of the virus for replication or produces a messenger RNA^10^. The consecutive aspartates active site were found and targeted in many viral RNA polymerases. Therefore, inhibition of RdRp prevents these essential steps in the lifecycle of SARS-CoV-2 and, thus, prevents replication and propagation of the virus.

Sofosbuvir is a uridine nucleotide analog that potently inhibits viral polymerases. It is an anti-RdRp that is found useful in treating SARS-CoV-2 patients^11^. Sofosbuvir is a protide prodrug that is hydrolyzed by liver enzymes after absorption to form the monophosphate uridine analog, which is further phosphorylated to form the active triphosphate form. Sofosbuvir was approved by FDA against hepatitis C Virus (HCV) in the year 2013. It is administered orally in combination with ribavirin to treat HCV of genotypes 2 and 3^12^. We choose this drug as it was deeply studied with millions of HCV patients worldwide during the past 10 years.

The SARS-CoV-2 RNA genome frequently mutates due to its rapid replication and lack of error proofreading by the viral RNA polymerase, which results in the emergence of drug resistance (e.g., the P323L mutation where the substitution of proline [Pro] to leucine [Leu] occurred at amino acid position 323 of RdRp)^13,14^. Therefore, continuous development of drugs is required to overcome emerging viral resistance.

**Figure 1:**
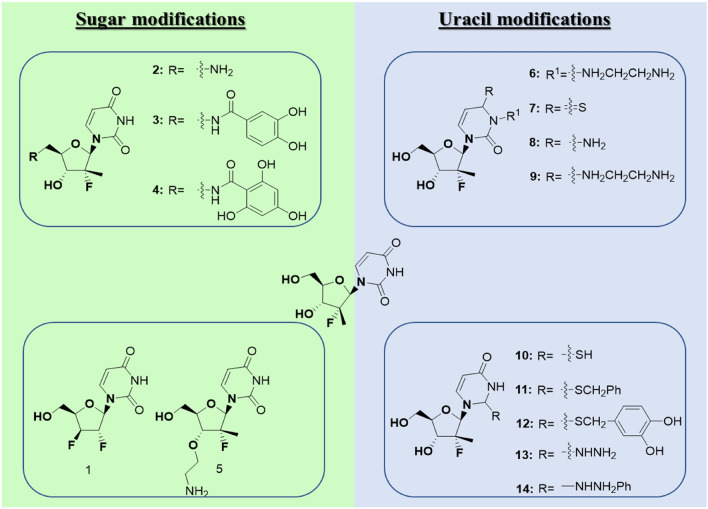
Sugar and uracil modifications to the sofosbuvir drug.

In this study, our strategy was to carry out rational modifications to the general features of the sofosbuvir scaffold and to model the effect of such alterations on RdRp binding. Modifications were categorized into two groups based on the site of transformation: sugar modifications and uracil modifications. Sugar modifications include studying the effect of extending position 3 with the ethyl amino group or changing position 3 to the fluoro group (structures 1 and 5, Fig. 1)^15^. To mimic the binding of the triphosphate, an amino group, or polyhydroxylated-substituted amino groups, substitutes the hydroxyl of the ribosyl 5-position. The uracil modifications of sofosbuvir include attaching the ethylamino group at uracil N-3 and substituting the carbonyl oxygen at position 4 with sulphur or amino or aminoethyl amino groups (structures 6 – 9, Fig. 1)^16^. Other modifications involve substitutions at the carbonyl oxygen at position 2 with thio, benzylthio, and dihydroxy benzylthio groups (structures 10 – 12, Fig. 1). The effect of attaching hydrazine or phenylhydrazine at position 6 was also explored (structures 13 and 14, Fig. 1)^17^. To evaluate the proposed modifications, all analogs were docked in the catalytic site of RdRp, and the molecular dynamics of the best hits were determined to study the stability of the analogs in the gorge of the enzyme.

Molecular dynamics simulation (MDS) was successful in repurposing and prediction of new therapeutics against SARS-CoV-2 proteins^18,19^. The RdRp was previously studied using the MDS technique and was successful in predicting the potential usefulness of many drugs such as remdesivir against COVID-19 pandemic^20–22^. In the current study we use the same technique to test new scaffold molecules based on sofosbuvir.

## Materials and methods

### Protein and ligands preparation

As previously reported, the PDB: ID 7BV2 was downloaded from the Protein Data Bank (www.pdb.org )^23^ and was then prepared by removing unwanted molecules and adding missing Hydrogen atoms using PyMOL software^24,25^. As a result, the prepared protein target contained only nonstructural protein 12, nonstructural protein 7, and nonstructural protein 8 as a complex, the active complex of the viral polymerase^26^. We choose this structure as it is of moderate resolution (2.5 Å) and containing the polymerase in conjunction with nsp7 and nsp12, representing the complete system.

The drug was modified and optimized using SCIGRESS 3.2 software^27^. First, the structures were minimized using the classical MM3 force field, followed by the semiempirical parameterization method (PM6) in water^28^. Next, the vibrations were calculated at the same level of computation to ensure their stability (not a transition state). Finally, structures were optimized using the quantum mechanical method, which is based on density functional theory (DFT), using the B3LYP functional^29,30^. Again, geometry was judged by their infra-red spectrum calculated at the same level of theory. We ensure no negative vibrations (transition states) are found in the optimized compounds.

### Molecular docking

The clustered trajectories (three clusters) obtained from the previous study were used as possible protein conformations for docking^24^. The clustering was performed using Chimera using default parameters. Prior to the simulation any water, ions, or ligands were removed. AutoDock Vina (7) was used for the flexible docking of the modified compounds with each cluster representative. The compounds were prepared by adding Gasteiger charges and converted to PDBQT file format using AutoDock Tools software^31^. The protein was also prepared by adding partial charges and converted to PDBQT file format. The grid box dimensions were set to 40 Å in each direction for each docking experiment to cover the active site residues. The box centers for the docking with cluster 1, cluster 2, and cluster 3 representative structures (after clustering the trajectories of the protein target) were set to (− 5.194, − 9.118, 0.000), (− 6.871, − 10.988, − 0.146), and (− 5.558, − 9.933, − 0.090), respectively. According to the calculated binding affinity, the best complex (having the lowest binding energy values) and the parent compound, sofosbuvir, were subjected to another MDS run of 100 ns. We compared the binding behaviour of the tested compounds to the reference drug (sofosbuvir) using the same protocol.

### Molecular dynamic simulation (MDS)

The files were prepared for MDS using the CHARMM-GUI web server^32,33^ (https://www.charmm-gui.org/). The system was solvated using the TIP3P water model in a cubic box with a padding of 10 Å and the box size was 121 × 121 × 121 Å^3^ then the systems were neutralized by adding 0.154 M NaCl which resulted in a total of around 166,000 Atoms. The temperature was set to the physiological value of 37 °C (310 K), and the CHARMM36 force field was utilized. CHARMM36 accurately predict protein parameters and is utilized by many MDS software when dealing with proteins. It could predict backbone & side-chain scalar couplings, residual dipolar couplings, and relaxation & side-chain order parameters^34^.

Nanoscale molecular dynamics (NAMD) 2.13 software was used to perform the MDS calculation^35^. Before the production run, the minimization and equilibration steps were completed. The system was minimized for 10,000 steps and then equilibrated for 0.25 ns in an ensemble of a constant number of atoms, a constant volume, and a constant temperature (NVT). The temperature was maintained using Langevin dynamics^35,36^. Then, the production run was started for 100 ns in the NVT ensemble. Finally, the MDS trajectories were analyzed using in-house codes and visualizing molecular dynamics (VMD) 1.9.3 software^37^. In addition, the trajectory of the best two complexes and the reference compound were clustered using TTClust and representative frames for each cluster were obtained. Protein–Ligand Interaction Profiler (PLIP) was used with these frames to detect the interacting amino acids^38–40^.

### Binding energy calculations during the molecular dynamics simulation for the best hits

MMPBSA.py module implemented in the AmberTools 21 package was utilized to determine the binding affinity for the best-hit compounds (**3** and **4**) and the positive control sofosbuvir^41,42^. In addition, decomposition analysis was calculated to determine the contribution of amino acids to total binding energy. The salt concentration and solvation method (igb) were set to 0.154 M and 5, respectively. The internal and external dielectric constants were set to 1.0 and 80.0, respectively, and other options were assigned to the default values. The molecular mechanics- generalized Born surface area (MM-GBSA) approach is depicted in Eq. (1).

1 $$\Delta {\text{G = < G}}_{{{\text{complex}}}} {\text{ - (G}}_{{{\text{receptor}}}} {\text{ + G}}_{{{\text{ligand}}}} {) > }$$

where <  > represents the average of the enclosed free energies of complex, receptor, and ligand over the frames used in the calculation. In our approach, we used the whole trajectory (1000 frames). Different energy terms can be calculated according to Eqs. (2) – (6) as follows:

2 $$\Delta {\text{G}}_{{{\text{binding}}}} { = }\Delta {\text{H - T}}\Delta {\text{S}}$$

3 $$\Delta {\text{H = }}\Delta {\text{E}}_{{\text{gas }}} { + }\Delta {\text{E}}_{{{\text{sol}}}}$$

4 $$\Delta {\text{E}}_{{\text{gas }}} { = }\Delta {\text{E}}_{{\text{ele }}} { + }\Delta {\text{E}}_{{\text{vdW }}}$$

5 $$\Delta {\text{E}}_{{{\text{solv}}}} {\text{ = E}}_{{\text{GB }}} {\text{ + E}}_{{{\text{SA}}}} { }$$

6 $$ESA = \gamma .SASA$$

where ∆H is the enthalpy calculated from gas-phase energy (E_gas_) and solvation-free energy (E_sol_), − T∆S is the entropy contribution to the free binding energy, and E_gas_ is composed of electrostatic term E_ele_ and van der Waals term E_vdW_. E_sol_ can be calculated from the polar solvation energy (E_GB_) and nonpolar solvation energy (E_SA_), which are estimated from the solvent-accessible surface area. The entropy contribution was calculated using the interaction entropy method using the last 90 ns^43,44^.

## Results and discussion

Sofosbuvir is a uridine nucleotide analog that potently inhibits HCV NS5B polymerase. Sofosbuvir is a protide prodrug that is hydrolyzed by liver enzymes after absorption to form the monophosphate uridine analog, which is further phosphorylated to form the active triphosphate form. Sofosbuvir received FDA approval for use against HCV in 2013; it is administered orally in combination with ribavirin for the treatment of genotypes 2 and 3^45^.

### Molecular docking

**Figure 2:**
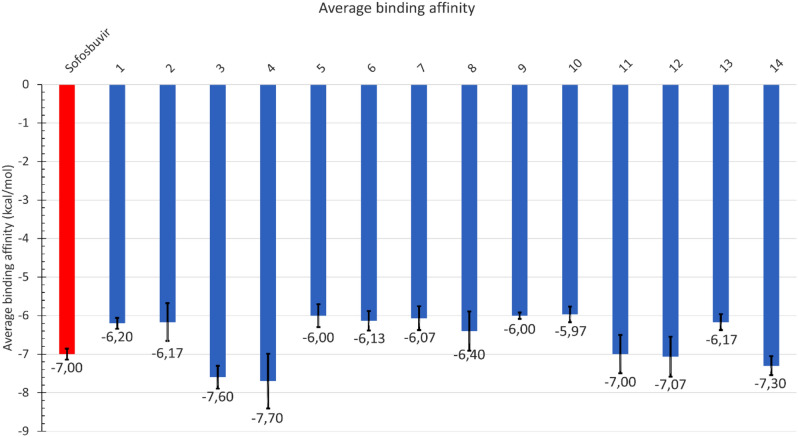
The average binding affinities (in kcal/mol) of sofosbuvir(red) and its fourteen modifications (blue) against SARS-CoV-2 RdRp.

The average docking scores of the suggested modifications of sofosbuvir (Fig. 1) and the positive control compound (sofosbuvir triphosphate), calculated with AutoDock Vina, are presented in the bar graphs in Fig. 2. The docking experiments were performed with the three different conformations of the SARS-CoV-2 RdRp after 100 ns MDS ^24^. The error bars represent the standard deviations. As shown in Fig. 2, the fourteen compounds showed average binding energies to the SARS-CoV-2 RdRp active site comparable to that of the positive control and parent compound, sofosbuvir. Additionally, five compounds (**3**, **4**, **11**, **12**, and **14**) showed better average values than sofosbuvir in binding SARS-CoV-2 RdRp, while compounds **3** and **4** were the best hits in binding the viral polymerase.

Table 1 summarizes the formed interactions that were established upon docking these fourteen compounds in the active site of the SARS-CoV-2 RdRp. Two types of interaction stabilized the complexes; Hydrogen bonding (H-bonds) and hydrophobic interactions. On average, 2.9 ± 1.8 H-bonds and 1.8 ± 0.8 hydrophobic contacts were established upon docking the fourteen ligands against the SARS-CoV-2 RdRp active site. In contrast, five H-bonds were formed for sofosbuvir, and only one hydrophobic contact was established upon docking. For the best two compounds, **3** and **4**, seven interactions were established between the ligands and the protein residues. Additionally, the following six residues were the most significant contributors in the fourteen compounds against the SARS-CoV-2 RdRp active site: D618 (8), D761 (5), A762 (7), W800 (5), E811 (4), and C813 (4), with the numbers in brackets, indicating the total number of interactions (as shown in Table 1, bolded residues). All these residues were found in the active site pocket of the RdRp, as previously reported^46^.

**Table 1:** Detailed interactions established upon docking the sofosbuvir and its derivatives (1–14) against SARS-CoV-2 RdRp in complex with NSP8 and NSP12 retrieved from PLIP webserver and PyMOL software.

Ligand	Hydrogen bonds		Hydrophobic interactions	
	No	Residues involved	No	Residues involved
Sofosbuvir	5	A550, K551, **W800**, H810, and C813	1	**D618**
1	3	**W800**, and C813(2)		
2	1	C813	2	**D618** and** D761**
3	6	S549, A550, K551, **W800**, C813, and R836	1	**D618**
4	7	K545, R555, Y619, R624, S759, **D760**, and **D761**		
5	2	**A762** and **W800**	1	**D618**
6	1	**A762**	1	**D618**
7	1	I548	3	I837, A840, and L862
8	3	**A762(2)** and **W800**	2	**D618** and **D761**
9	3	**A762**, H810, and R836	2	**D618** and **E811**
10	2	**W800** and R836	1	**D761**
11	2	S814 and R858	3	I548, R836, and D865
12	3	R555 and **A762(2)**	1	**D618**
13	5	W617, K798(2), C799, and **E811**	3	**D618**, **D761**, and **E811**
14	2	K551 and K798	1	**E811**

Bold residues indicate the residues most reported to form interactions with the ligands.

### Molecular dynamics of the sofosbuvir- and the best compounds -RdRp complexes

**Figure 3:**
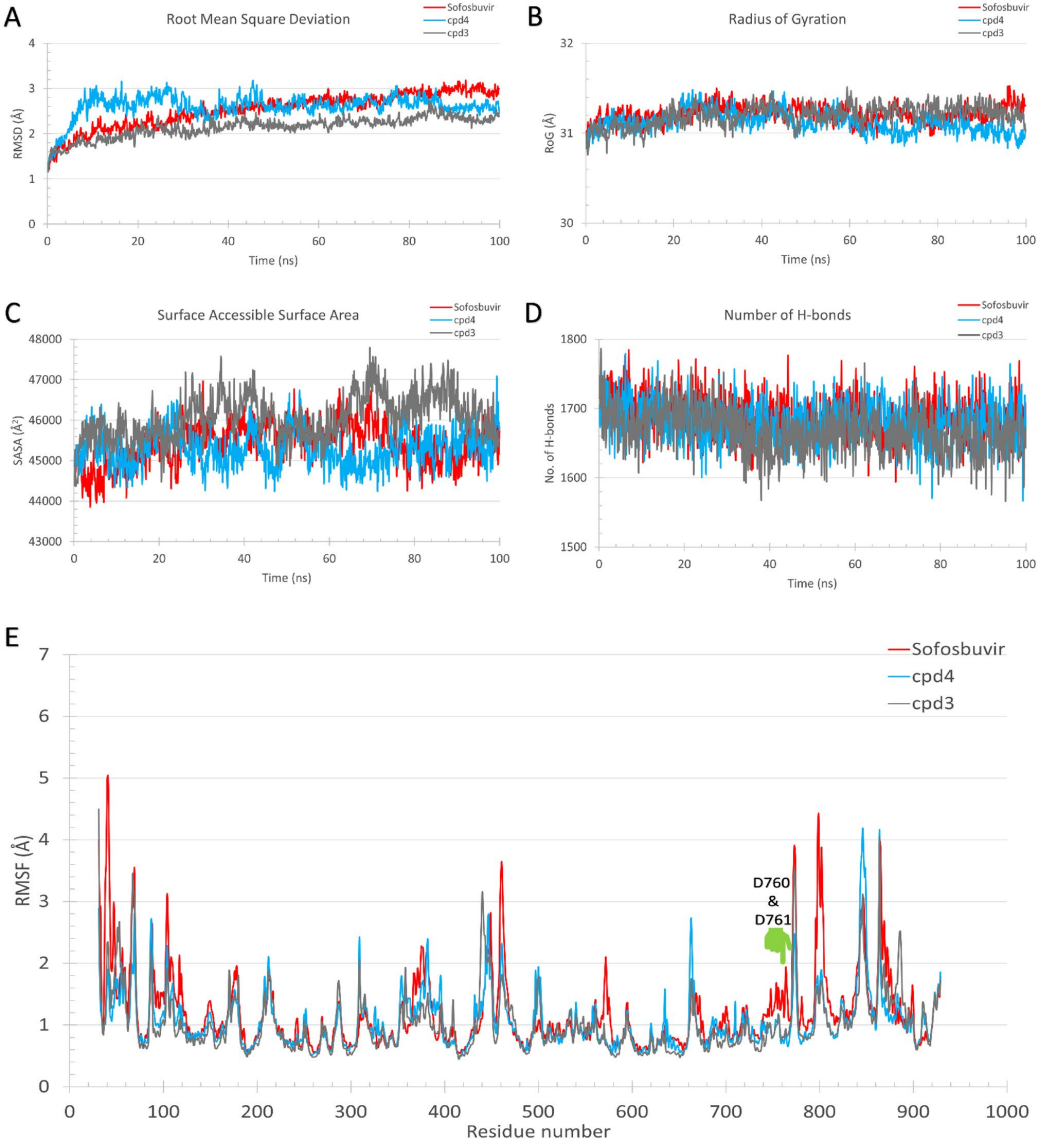

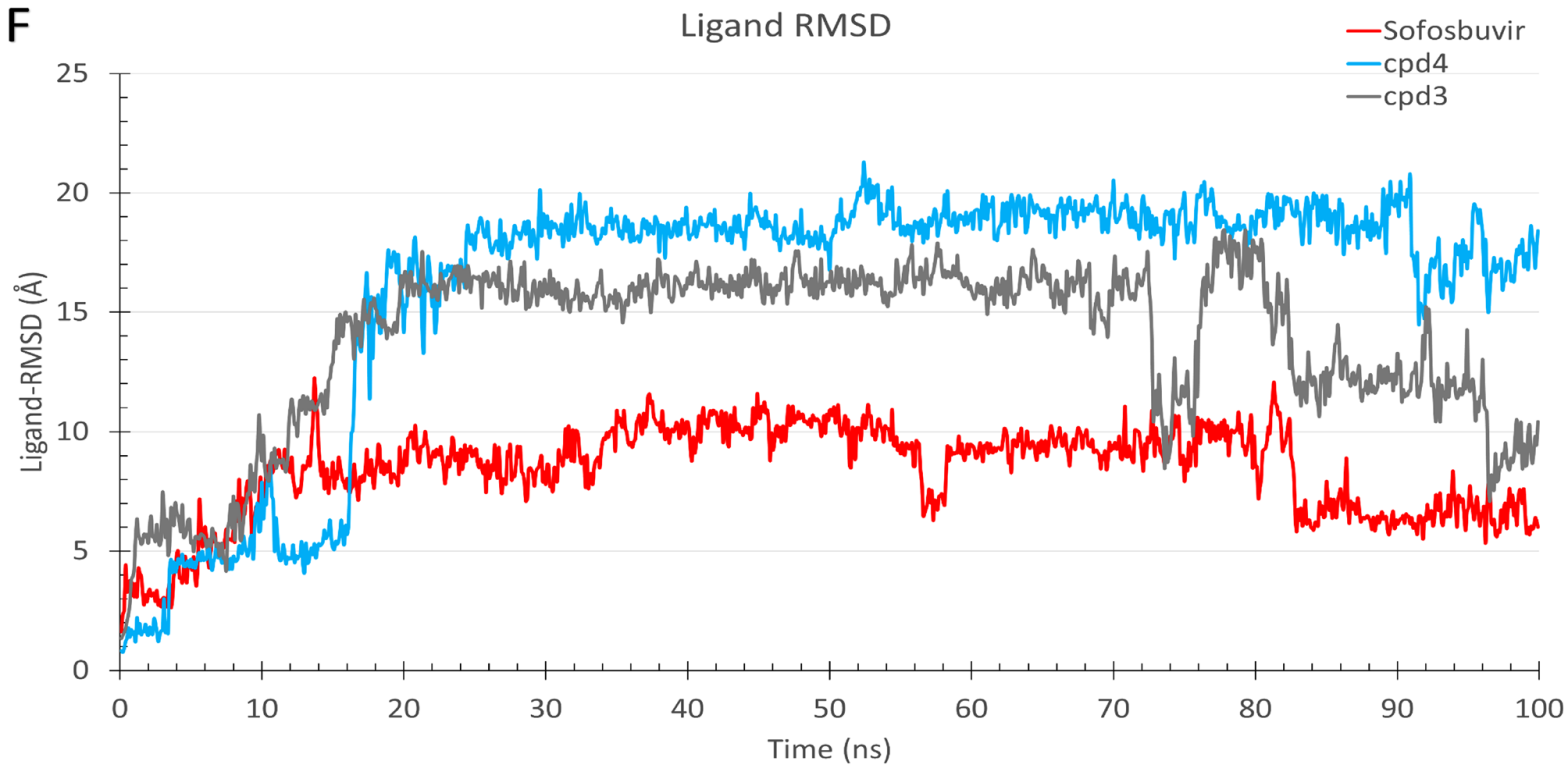
The molecular dynamics simulation analysis of the three best-hit complexes (4-RdRp) and the three sofosbuvir-RdRp complexes. (**A**), (**B**), (**C**), and (**D**) are the root-mean-square deviation (RMSD), the radius of gyration (RoG), the surface accessible surface area (SASA), and the total number of formed H-bonds versus the simulation time in ns. (**E**) The pre-residue root-mean-square fluctuations (RMSF) of the six complexes during the simulation period. The position of active site residues (D760 and D761) is marked in green. (**F**) The ligand RMSD versus the simulation time in ns.

MDS was used to assess protein system stability and to assess the binding affinities during system equilibration^47^. We simulated the protein–ligand complexes for both sofosbuvir (positive control) and compounds **3** and **4** for 100 ns MDS each. Figure 3 shows the root-mean-square deviation (RMSD) in Å (A), the radius of gyration (RoG) in Å (B), the surface accessible surface area (SASA) in Å^2^ (C), and the total number of formed H-bonds (D) versus the simulation time in nanoseconds. Sofosbuvir-RdRp complexes are shown in red, while compounds **3** and **4**-RdRp complexes are shown in gray and blue, respectively. Based on the RMSD curves (Fig. 3A), all the complexes were stable, and the systems were slowly equilibrated during the simulation period with RMSD values between 2 and 3.2 Å during the second half of the simulation. Additionally, the systems were stable during the simulation with RoG values around 31 Å (Fig. 3B), SASA values between 44,000 and 47,000 Å^2^ (Fig. 3C), and total system H-bonds between 1600 and 1750 (Fig. 3D). The per-residue root-mean-square fluctuations (RMSF) in Å are shown in Fig. 3E. The active site residues, which are marked on the RMSF curve, were stable (RMSF > 2Å). In general, the RMSF showed stable protein with minimal high fluctuation regions (RMSF < 3Å), as reported previously^48^. This indicates the stability of the complexes during the simulation period. The ligand-RMSD (Fig. 3F) also indicate stable complexes during the simulation period.

**Figure 4:**
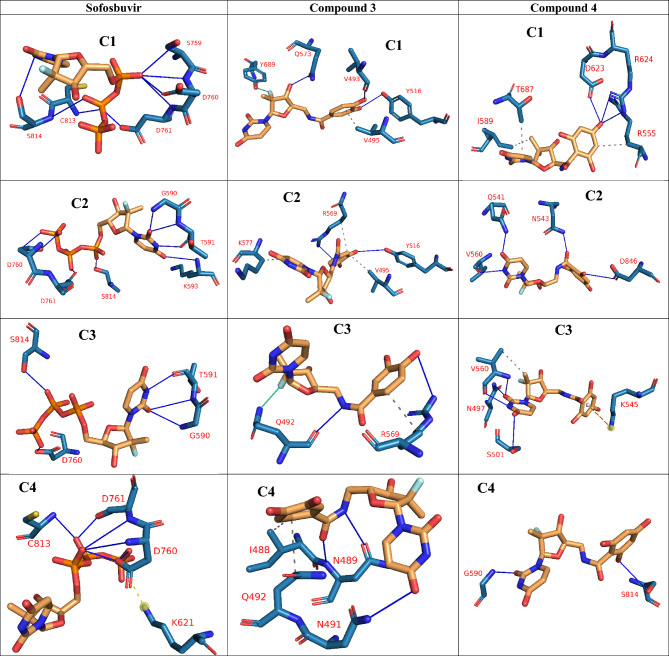
Shows the interactions detected using PILP webserver (https://plip-tool.biotec.tu-dresden.de/plip-web/plip/index) and then depicted using PyMOL 2.0.4 software on the representative frames obtained from the clustering of each trajectory after MDS. H-bonds are shown in blue lines, while hydrophobic contacts are shown in dashed-grey lines.

The Fig. 4 shows the results of trajectory clustering and the interacting amino acids as detected by PLIP. As can be seen in the representative frames of sofosbuvir, D760 and D761 are nearly common in all representative frames and forming three to four H-bonds. Moreover, K621 forms a salt bridge with sofosbuvir. For compound 3, the first two cluster representatives show V495 and Y516 forming one hydrophobic contact and one H-bond, respectively. In addition, the Fluorine atom form a halogen bond with Q492. For compound 4, there is no common amino acids in the representative frames, however, K545 forms a π-cation interaction.

The binding energies of the top hits’ (compounds **3** and **4**) complexes with RdRp were compared to the sofosbuvir-RdRp complexes during the simulation period using the molecular mechanics- generalized Born surface area (MM-GBSA) method. All the equilibrated region of trajectories (the last 90 ns) were used for the MM-GBSA calculations (stride = 1), and the values are listed in Table 2. The residual contribution to binding is listed in the table, where highly contributed residues (binding energy > -0.85 kcal/mol) are shown in bold. In all complexes, the ligand (LIG) was the most contributed element for the binding with values between − 7.78 and − 10.10 kcal/mol. The average total binding energy for the compounds **3**- and **4**-RdRp complexes (− 16.28 ± 5.69 and − 16.25 ± 5.78 kcal/mol) is comparable to that of the average binding energy of sofosbuvir-RdRp complexes (− 16.20 ± 6.35 kcal/mol). However, the entropy contribution of the reference compound is higher than that of the two proposed compounds. This indicates the feasibility of using these candidates as anti-SARS-CoV-2 RdRp after toxicity testing and experimental validation.

**Table 2:** The MM-GBSA calculations for sofosbuvir and the best hits (compounds 3 and 4) complexes with SARS-CoV-2 RdRp after 100 ns MDS. Bold residues indicate the most contributed residues (binding energy <  − 0.85 kcal/mol), while red-colored residues have a negative contribution to the binding (positive binding energies). The average binding free energies and their individual terms are shown at the bottom for each complex, along with its standard deviations. Entropy (− TΔS) was calculated using the interaction entropy using the last 90 ns of each trajectory.

RDRP complex	Sofosbuvir		Compound 3		Compound 4	
	Residue	Binding energy (kcal/mol)	Residue	Binding energy (kcal/mol)	Residue	Binding energy (kcal/mol)
Residual contribution to binding	**LIG**	** − 10.10103**	**LIG**	** − 7.78**	**LIG**	** − 8.49**
	**C733**	** − 2.04689**	**R489**	** − 1.66**	**V477**	** − 0.88**
	**D680**	** − 1.21198**	**Q493**	** − 1.1**	L678	− 0.35
	**L678**	** − 0.98482**	**V415**	** − 0.85**	D543	− 0.34
	**S734**	** − 0.908**	K497	− 0.31	C733	− 0.34
	I509	− 0.56127	L418	− 0.30	K465	− 0.33
	S679	− 0.51494	T485	− 0.18	I509	− 0.32
	R475	− 0.31803	L496	− 0.13	R475	− 0.18
	A608	− 0.26359	Y436	− 0.08	A478	− 0.15
	F732	− 0.25081	A500	− 0.08	G603	− 0.15
	N611	− 0.22928	S421	− 0.05	S679	− 0.15
	Q735	− 0.11987	N488	− 0.05	G510	− 0.14
	T607	− 0.11078	N417	− 0.05	N463	− 0.13
	D543	+ 0.08207	I509	− 0.05	S602	− 0.12
	E731	+ 0.19694	N416	− 0.04	A467	− 0.09
	D538	+ 0.21456	R574	+ 0.01	K541	+ 0.03
	K465	+ 0.35558	E578	+ 0.02	D680	+ 0.13
	D681	+ 0.44948	D419	+ 0.03	D681	+ 0.14
ΔE_VDW_ *(kcal/mol)*	− 19.84 ± 5.33		− 25.55 ± 7.08		− 26.88 ± 5.98	
ΔE_ELE_ *(kcal/mol)*	− 118.75 ± 26.37		− 10.18 ± 8.60		− 24.25 ± 13.21	
ΔG_GB_ *(kcal/mol)*	125.85 ± 22.72		23.19 ± 7.60		38.75 ± 12.74	
ΔG_SA_ *(kcal/mol)*	− 3.46 ± 0.58		− 3.74 ± 0.94		− 3.87 ± 0.88	
ΔG_GAS_ *(kcal/mol)*	− 138.59 ± 24.01		− 35.73 ± 9.79		− 51.13 ± 16.01	
ΔG_SOLV_ *(kcal/mol)*	122.39 ± 22.80		19.44 ± 7.44		34.88 ± 12.18	
-TΔS *(kcal/mol)*	104.2 ± 13.21		29.27 ± 1.94		37.31 ± 4.89	
ΔG total *(kcal/mol)*	** − 16.20** ± 6.35		** − 16.28** ± 5.69		** − 16.25** ± 5.78	

### ADMET prediction

With the help of the pkCSM web server^49^, the ADMET (Absorption, Distribution, Metabolism, Excretion, and Toxicity) properties of the three compounds were predicted. Table 3 shows the predicted values for each property. The three compounds show similar water solubility (log(mol/L)), with compound 4 being the more soluble one (− 2.84 log(mol/L)), followed by compound 3 (− 2.524 log(mol/L)), and then sofosbuvir (− 2.271 log(mol/L)), which are in the same region as other druggable compounds^50^. On the other hand, the Caco2 permeability shows a similar trend, with compound 4 as the most permeable (58.2%), followed by compound 3 (49.9%), and then sofosbuvir (28.7%).

**Table 3:** The ADMET predictions performed by pkCSM web server.

	Sofosbuvir	Compound 3	Compound 4
Absorption			
Water solubility	− 2.271	− 2.524	− 2.84
Caco2 permeability	0.287	0.499	0.582
Distribution			
Fraction unbound (human)	0.451	0.292	0.291
BBB permeability	− 2.577	− 1.38	− 1.647
CNS permeability	− 3.974	− 4.083	− 4.886
Metabolism			
CYP1A2 inhibitor	No	No	No
CYP2C19 inhibitor	No	No	No
CYP2C9 inhibitor	No	No	No
CYP2D6 inhibitor	No	No	No
CYP3A4 inhibitor	No	No	No
Excretion			
Renal OCT2 substrate	No	No	No
Toxicity			
AMES toxicity	No	No	No
hERG I inhibitor	No	No	No
hERG II inhibitor	No	No	No
Hepatotoxicity	No	Yes	Yes

The fraction unbound indicates the predicted fraction of compound that will be unbound to serum proteins. High unbound fraction, low blood–brain barrier (BBB) permeability (log BBB), and low central nervous system (CNS) permeability (log CNS) indicate a good distribution and low permeability to the BBB and CNS. All three compounds show low BBB permeability as they have values <  − 1 and low CNS permeability (values <  − 3). However, the fraction unbound shows a better value for the reference compound in comparison with the two compounds.

Inhibitors of Cytochrome P450 can activate the drug metabolism and, therefore, can remove the compound from the market. All compounds were predicted to not act as inhibitors of different isoforms (CYP1A2, CYP2C19, CYP2C9, CYP2D6, and CYP3A4).

For excretion, the model predicts that all compounds are not substrates for renal organic cation transporter 2. The interaction with this transporter helps in the clearance of the compound and may produce adverse interactions; therefore, a negative prediction is considered good.

Finally, four indicators are used to predict the toxicity of the compounds. AMES toxicity is a test that indicates whether the compound is a carcinogen. Inhibition of hERG I/II is the principal cause of fatal ventricular arrhythmia and has resulted in the withdrawal of many substances. As its name implies, hepatotoxicity indicates whether the compound may disrupt the normal function of the liver. The server predicted that all compounds do not act as carcinogens or inhibitors for hERG I/II. On the other hand, the two compounds were predicted to have a hepatotoxic effect, while the reference does not.

## Conclusion

SARS-CoV-2 RdRp is a well-known viral protein target for drug designers. It has been studied for possible inhibition by previously approved drugs, such as remdesivir and sofosbuvir. In this study, we tested 14 novel modifications of sofosbuvir against the RdRp of SARS-CoV-2 in silico. Our docking study revealed the potential of five modifications (compounds **3**, **4**, **11**, **12**, and **14**), as they obtained binding comparable to that of the parent compound, sofosbuvir, against the RdRp active site. Additionally, the molecular dynamics simulation revealed the effectiveness of compounds **3** and **4** as the best RdRp binders. It form stable complexes with SARS-CoV-2 RdRp mainly through V415, V477, R489, and Q493 with binding energies of − 16.28 and − 16.25 for compounds 3 and 4, respectively. This study paves the way for the laboratory verification of for novel SARS-CoV-2 RdRp blockers.

## Data Availability

Data is available upon request from the corresponding author.
